# Development and Validation of a Forensic Multiplex System With 38 X-InDel Loci

**DOI:** 10.3389/fgene.2021.670482

**Published:** 2021-08-17

**Authors:** Ling Chen, Xiyong Pan, Yuan Wang, Weian Du, Weibin Wu, Zhenya Tang, Cheng Xiao, Xiaolong Han, Chao Liu, Changhui Liu

**Affiliations:** ^1^School of Forensic Medicine, Southern Medical University, Guangzhou, China; ^2^School of Forensic Medicine, Sun Yat-sen University, Guangzhou, China; ^3^Guangdong Homy Genetics Incorporation, Foshan, China; ^4^AGCU ScienTech Incorporation, Wuxi, China; ^5^Guangdong Provincial Forensic Science Center, Guangzhou, China; ^6^Guangzhou Forensic Science Institute, Guangzhou, China

**Keywords:** InDel, X chromosome, multiplex PCR, validation, forensic genetics

## Abstract

In the present study, a novel multiplex system, AGCU X-InDel 38 kit, was designed to amplify 38 X-InDel markers and amelogenin in a single Polymerase Chain Reaction (PCR). To demonstrate the suitability and efficiency for forensic applications, a series of validation experiments were conducted, including sensitivity, species specificity, reproducibility, stability, case samples, balance of peak height, size precision, as well as allele frequency and forensic parameter analysis. The results showed that AGCU X-InDel 38 kit was capable to get full profiles even with 62.5 pg of template DNA, and full profiles can be obtained when hematin concentration ≤25 μmol/L, or hemoglobin concentration ≤50 μmol/L, showing good tolerance to six common inhibitors. Moreover, the analyzed case samples indicated that AGCU X-InDel 38 kit had better performance for degraded and trace DNA samples. The 200 unrelated males from Guangdong Han population showed that the combined PD_Male_ and PD_Female_ were both more than 0.999999999, and the combined MEC_Krüger_, MEC_Kishida_, and MEC_Desmarais  Duo_ were 0.999369481, 0.999999917, and 0.999941556, respectively. Robust discrimination capability of this novel multiplex system could be demonstrated through the high values of forensic parameters. In conclusion, AGCU X-InDel 38 kit is sensitive, precise, reproducible, and highly informative and could be used as a complementary tool for complex and challenging kinship cases.

## Introduction

Short tandem repeat (STR) loci represent the mainstream markers for forensic investigations. However, with the large-scale application of STR typing techniques, its disadvantages have been gradually exposed, such as high mutation rates and poor performance for degraded DNA (Zhang et al., 2009; Ip et al., 2014; Siriboonpiputtana et al., 2018; Xiao et al., 2018). In addition, autosomal STRs may not be effective in some deficiency paternity cases. Taking advantages of X-linked inheritance into consideration, X chromosome markers are of great value in some specific and deficient kinship cases especially when autosomal chromosome markers are uninformative, for instance, incest cases of father–daughter, some other paternity cases of grandmother–granddaughter as well as half-sisters (Szibor, 2007; Tillmar et al., 2017; Gomes et al., 2020). Different from STRs, InDel markers have a short amplicon size and low mutation rates, and simple analytical procedures are required (LaRue et al., 2012; Chen et al., 2019). In recent years, there is a growing tendency in studying X chromosomal InDel markers, especially in the field of evolutionary anthropology, assessing admixture of population and kinship investigations with deficient relationship (Ribeiro-Rodrigues et al., 2009; Resque et al., 2010; Ibarra et al., 2014).

Though a few multiplex amplification systems about X-InDel loci have been developed, such as 33 plex X-InDel system (Freitas et al., 2010), 32 plex X-InDel system (Pereira et al., 2012), and 18 plex X-InDel system (Zhang et al., 2015), 21 plex X-InDel system (Edelmann et al., 2016), there is no commercial X-InDel kits until now. In this study, we developed a novel five-dye X-chromosome InDel typing system, AGCU X-InDel 38 kit, which comprises 38 highly polymorphic X-InDel loci and one amelogenin gene locus. Expected as a supplementary tool for some complex and challenging kinship cases, especially for those degraded DNA samples, AGCU X-InDel 38 kit is characterized by smaller amplicon sizes, more loci, and higher cumulative discriminatory power. Before forensic application and commercialization of this kit, we conducted this study to evaluate its suitability and efficiency. All the studies were carried out under the developmental validation guidelines of the Scientific Working Group on DNA Analysis Methods (SWGDAM), which comprised sensitivity, species specificity, reproducibility, stability, case samples, balance of peak height, size precision, population allele frequency, and forensic parameter analysis.

## Materials and Methods

### Marker Selection and Primer Design

According to sequences from the dbSNP database, X-InDel markers were selected to meet the following criteria: (1) the difference of allelic X-InDel fragment in length, the inserted or deleted base number in other words, ranged from 1 to 25 bp; (2) all loci were located in introns; (3) the minor allele frequency (MAF) varied from 0.4 to 0.5 in East Asians; and (4) all loci comply with the Hardy–Weinberg equilibrium in the Chinese population. Finally, we got 38 optimal X-InDel markers, among which four multi-InDel markers (rs59605609, rs79829945, rs143123845, and rs35574346) with more than two alleles were involved in order to improve the discrimination power. Besides, the amelogenin gene was added for the design of a sex-confirmation marker. The information about the 38 selected X-InDel loci and amelogenin marker are shown in Table 1.

**TABLE 1:** The information of 38 X-InDel loci and amelogenin presented in the AGCU X-InDel 38 kit.

**Locus**	**Chromosome localization**	**Label**	**Allelic ladder**	**9948 genotypes**
Amel	Xp22.1-22.3,Yp11.2	FAM	X,Y	X,Y
rs3859989	Xq23	FAM	D,I	I
rs57608175	Xq24	FAM	D,I	I
rs4030406	Xp11.23	FAM	D,I	I
rs3216913	Xp22.2	FAM	D,I	D
rs2308280	Xp21.3	FAM	D,I	D
rs59605609	Xq24	FAM	D1I2,I1D2,I1I2	I1I2
rs56820033	Xq22.2	FAM	D,I	D
rs79829945	Xp22.2	FAM	D1D2,I1I2	I1I2
rs16397	Xq25	HEX	D,I	I
rs61260787	Xq23	HEX	D,I	D
rs2307741	Xq28	HEX	D,I	D
rs25581	Xp22.2	HEX	D,I	I
rs143123845	Xp22.31	HEX	I, I1I2	I
rs10699224	Xp22.2	HEX	D,I	D
rs1160845	Xq25	HEX	D,I	D
rs2308033	Xq26.1	HEX	D,I	I
rs57843641	Xq26.1	HEX	D,I	D
rs3077884	Xq28	TAM	D,I	D
rs11277082	Xq22.1	TAM	D,I	I
rs71671860	Xq21.2	TAM	D,I	D
rs35574346	Xp11.4	TAM	D1I2,I1D2,I1I2	I1D2
rs34763847	Xq21.2	TAM	D,I	D
rs149102585	Xq23	TAM	D,I	D
rs16367	Xq22.2	TAM	D,I	D
rs10671504	Xp22.31	TAM	D,I	I
rs58595330	Xp22.2	TAM	D,I	D
rs16637	Xp11.23	ROX	D,I	I
rs36094418	Xp22.2	ROX	D,I	D
rs17394	Xq26.1	ROX	D,I	I
rs2307707	Xq21.32	ROX	D,I	D
rs16368	Xq22.1	ROX	D,I	I
rs3215490	Xq21.2-21.32	ROX	D,I	I
rs60283667	Xq21.3	ROX	D,I	D
rs3048996	Xp22.31-22.13	ROX	D,I	I
rs35954471	Xp22.31	ROX	D,I	D
rs363794	Xq21.33	ROX	D,I	D
rs45449991	Xq21.2	ROX	D,I	D
rs199731653	Xq21.3	ROX	D,I	I

The Polymerase Chain Reaction (PCR) primers corresponding to the InDel loci and amelogenin locus were filtered and obtained using the Oligo 6.0 software (Premier Biosoft International, Palo Alto, CA, United States). All primers were chosen based on the following requirements: (1) primer pairs had similar melting temperatures (Tm) to be amplified at a similar efficiency, (2) without self-complementarity, hairpin structure, and non-specific amplification, (3) BLAST was used as an alignment algorithm to design target-specific primer sequences, and (4) SNP locations were excluded in primer sequences for a successful amplification experiment.

### Making Allelic Ladders

#### Amplification and Purification of the Common Alleles in the Population

Genomic DNA that came from blood samples was amplified using non-fluorescent-labeled primers. PCR products were subsequently prepared for electrophoresis on a 1.7% agarose gel with 0.5 μg/ml ethidium bromide.

#### Monocloning of the Target Alleles

The PCR products were cloned into pMD18-T vectors and transferred into *Escherichia coli DH5*α overnight in Luria-Bertani medium. Then the recombinant plasmids were screened with the method of blue-white spot screening, extracted, and sequenced subsequently. The last, the plasmids were frozen, and the cultured bacteria were stored in glycerol for further use.

#### Allele Preparation

After the PCR amplification of those recombinant plasmids, each of single-locus ladders was prepared using all the allelic PCR products from the same locus. The peak height ratios of PCR products were detected by electrophoresis and adjusted through changing the concentration. All the allelic PCR products of the same locus were mixed, and their peak height ratio was kept in balance by adjusting the concentration. According to the average peak height ratio of each single-locus ladder, the allelic ladder of AGCU X-InDel 38 kit was prepared by adjusting the volume ratio of all ladders to ensure that their average peak height ratio was more than 0.85. All ladders were stored in the dark at −20°C.

#### Allelic Ladder Evaluation

The peak height of each allele usually exceeded 400 relative fluorescence units (RFUs). The allele peak height ratio of the loci was more than 70%. The average of peak height ratio between loci labeled with the same fluorescence dye was more than 50%, and the average of peak height ratio between loci labeled with different fluorescence dyes was more than 50%.

### DNA Amplification

After optimization, PCR amplification was performed in a 25.0-μl reaction volume comprising 10.0 μl of 2.5 × PCR buffer mixture (consisting of 125 mmol/L Tris-HCl buffer, 125 mmol/L KCl, 7.5 mmol/L dNTPs, 5 mmol/L MgCl_2_, 2 mg/ml BSA, and 1% PCR intensifier), 5.0 μl of 5 × primer mix, 1.0 μl of 5 U/μl heat-activated Taq polymerase (AGCU ScienTech Incorporation) and 0.5–2 ng of template DNA. sdH_2_O was adjusted to keep the total volume at 25.0 μl.

The PCR cycling conditions were as follows: initial denaturation at 95°C for 2 min, annealing elongation of 94°C for 30 s, 58.5°C for 1 min, and 72°C for 1 min for 29 cycles, final extension of 72°C for 10 min, and then hold at 4°C.

### Electrophoresis and Analysis

Following amplification, 1 μl of amplified product was added to 10 μl of Hi-Di^TM^ formamide and 0.3 μl of AGCU Marker SIZ-500 size standard, followed by being centrifuged for 3 min at 3,000 rpm. The mixture was denatured at 95°C for 3 min and then chilled on ice for 3 min. Subsequently, using the Applied Biosystems 3130XL Genetic Analyzer, fragments were separated with filter of E5 and polymer of POP-4 (Thermo Fisher Scientific, South San Francisco, CA, United States) by capillary electrophoresis, under the following parameters: injection at 2 kV for 10 s and electrophoresis at 15 kV for 1,500 s, 60°C. The raw CE data were genotyped by GeneMapper IDX 1.3 (Life Technologies).

### Developmental Validation Studies

#### Sensitivity

Control DNA 9947A (Promega, Madison, WI, United States) was selected as the template and performed with a dilution series at 1.0 ng, 0.5 ng, 0.25 ng, 0.125 ng, 62.5 pg, and 31.25 pg. Each input DNA was tested in a final volume of 10 μl and was repeated three times in parallel.

#### Species Specificity

Non-human DNA samples were extracted from *Gallus*, *Bovine*, *Canis*, *Anatidae*, *Osteichthyes*, *Muroidea*, *Suidae*, *Leporide*, and *Ovis* (which had been collected by Guangzhou Forensic Science Institute over years), respectively.

#### Reproducibility

Thirty samples from 200 Han population samples were used for simultaneous amplification detection and validation of genotyping results in three separate laboratories (Guangzhou Forensic Science Institute and two of its branches).

#### Stability

PCR inhibitors of hematin, hemoglobin, indigo, humic acid, EDTA, and calcium ion were added to 0.5 ng of control DNA 9948 (Promega, Madison, WI, United States) with the following concentration gradients: 25, 50, 75, 100, or 150 μmol/L of hematin; 50, 75, 100, 150, or 200 μmol/L of hemoglobin; 4, 8, 12, 16, or 20 mmol/L of indigo; 25, 50, 75, 100, or 150 mg/L of humic acid; 0.3, 0.6, 0.9, 1.2, or 1.5 mmol/L of EDTA; and 0.4, 0.8, 1.2, 1.6, or 2.0 mmol/L of calcium ion.

#### Case Samples

Fifty-six samples were collected from forensic cases comprising different types of biological specimens including bloodstain (*n* = 7), straw (*n* = 3), toothbrush (*n* = 3), cigarette butt (*n* = 4), swab of bottleneck (*n* = 5), buccal swab (*n* = 1), facial tissue (*n* = 4), touch DNA samples (*n* = 22), and formalin-fixed muscular tissue samples (*n* = 7). All samples above were detected by AGCU X-InDel 38 kit for the comparison with AGCU X19 STR kit.

#### Balance of Peak Height

A total of 80 samples from 200 Guangdong Han population were used to analyze the balance of peak height. The ratio of the minimum RFU to the maximum RFU for different loci and the mean peak heights for each group of loci were acquired from the mean value.

#### Size Precision

To demonstrate the size precision, allelic ladder was evaluated by three separate injections on a 3130XL Genetic analyzer, followed by calculating the average fragment size and the standard deviation of each allele.

#### PCR Conditions

Appropriate annealing temperature, and concentrations of primers and C-Taq polymerase are the guarantee of consistent and robust results in various laboratories. Too high or low may lead to non-specific PCR products, as well as lower PCR amplification efficiency (Rychlik et al., 1990). Under the conditions of different concentrations of InDel primers (0.5, 1, 2, 3, 4, 5, 6, 7, 8, 9, and 10 μmol/L), reaction mix (1/10, 2/10, 3/10, 4/10, 5/10, 6/10, 7/10, and 8/10), C-Taq polymerase (0.1, 0.2, 0.3, 0.4, 0.5, 0.6, 0.7, 0.8, 0.9, and 1.0 μl), and different annealing temperatures (55.9, 56.5, 57.2, 57.9, 58.5, 59.2, 59.9, and 60.5°C), 0.8 ng of control DNA 9948 was amplified using AGCU X-InDel 38 kit to determine the optimal PCR conditions.

#### Allele Frequency and Forensic Parameter Analysis

DNA samples of 200 individuals from Guangdong Han population were examined by AGCU X-InDel 38 kit after obtaining the informed consent. Basic statistical computations about forensic genetic parameters were performed with the StatsX v2.0 (Lang et al., 2019), including allelic frequencies, gene diversity (GD), polymorphic information content (PIC), power of discrimination in females (PD_Female_) and in males (PD_Male_), mean exclusion chance for deficiency cases (MEC_Krüger_), mean exclusion chance for normal trios (MEC_Kishida_), and mean exclusion chance for duo cases (MEC_Desmarais  Duo_). Tests of linkage disequilibrium (LD) were analyzed by Arlequin v3.5 software (Excoffier and Lischer, 2010).

### Quality Control

All experiments were performed according to the recommendations for the use of nomenclature and the guidelines for quality control as well as statistical analysis proposed by the SWGDAM and the International Society of Forensic Genetics (ISFG) (Schneider, 2007).

## Results and Discussion

### Sensitivity

**FIGURE 1:**
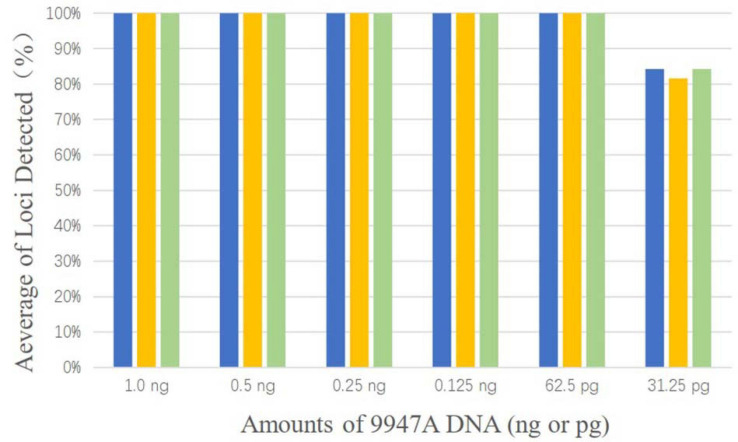
The detection ratios of AGCU X-InDel 38 kit in the sensitive study in triplicate (The threshold for detection was set at 50 RFU).

As shown in Figure 1, complete profiles were acquired when the template DNA concentration was higher than or equal to 62.5 pg. Allele dropouts were observed when template DNA inputs were further reduced to 31.25 pg. Therefore, the sensitivity of AGCU X-InDel 38 kit is 62.5 pg/10 μl.

### Species Specificity

The results of species specificity testing showed that no specific amplification products were observed (Supplementary Figure 1). Therefore, the genotyping results of AGCU X-InDel 38 kit were not affected by other common non-human DNA.

### Reproducibility

The results indicated that consistent allele calls were obtained consistently for all studied samples. The electropherograms of allelic ladder from three independent labs are shown in Supplementary Figure 2.

### Stability

**FIGURE 2:**
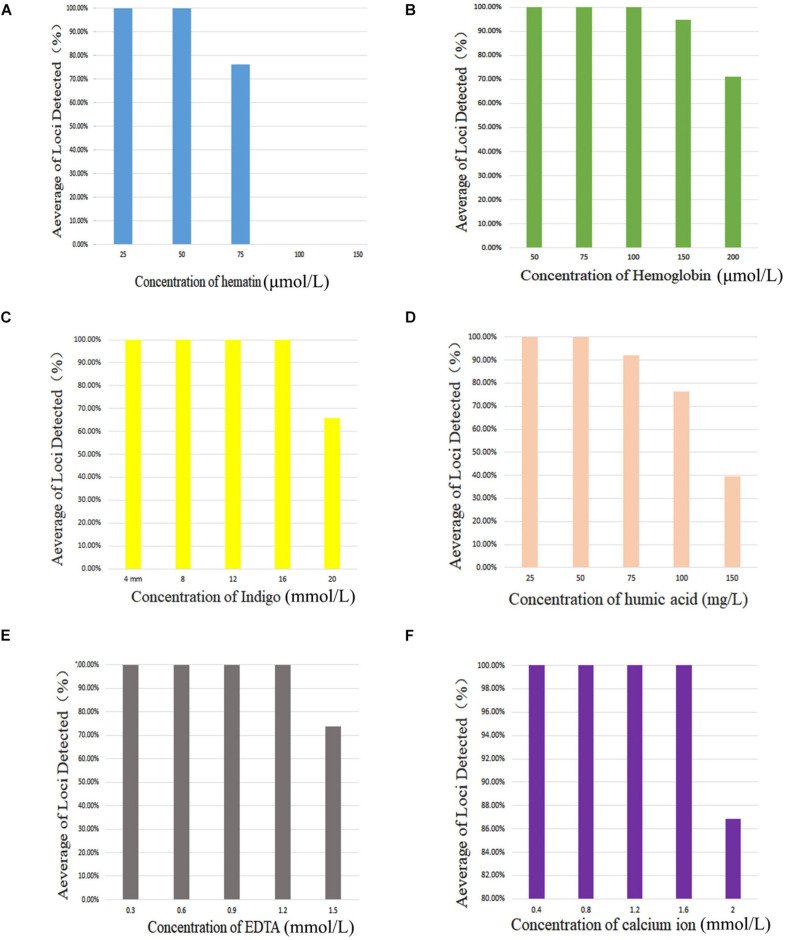
Average of loci detected with AGCU X-InDel 38 kit with increasing concentration of PCR inhibitors. **(A)** Amplification results at different concentrations of hematin, **(B)** hemoglobin, **(C)** indigo, **(D)** humic acid, **(E)** EDTA, and **(F)** calcium ion.

Figure 2 shows that complete profiles were obtained when amounts of inhibitors were not higher than 50 μmol/L hematin, 100 μmol/L hemoglobin, 16 mmol/L indigo, 50 mg/L of humic acid, 1.2 mmol/L EDTA, or 1.6 mmol/L calcium ion. Furthermore, once the amounts of inhibitors increased to 75 μmol/L hematin, 150 μmol/L hemoglobin, 20 mmol/L indigo, 75 mg/L humic acid, 1.5 mmol/L EDTA, and 2.0 mmol/L calcium ion, allele dropouts were observed. Those results revealed that this X-InDel system could tolerate considerable concentrations of six common inhibitors.

### Case Samples

As shown in Supplementary Table 1, the odds of obtaining a more complete DNA profile were found to be greater using AGCU X-InDel 38 kit than that using AGCU X19 STR kit. For example, complete profiles were obtained using AGCU X-InDel 38 kit from two samples of a 13-year-old bloodstain, while AGCU X19 STR kit only detected 10, 14 of 19 loci, respectively. Additionally, for those specimens that had been fixed in formalin for a long time, the AGCU X-InDel 38 kit was capable of getting complete profiles successfully, but the AGCU X19 STR kit could not. However, as for muscular tissue fixed in formalin, ratios of detected loci for both AGCU X-InDel 38 kit and AGCU X19 STR kit decreased significantly until immersion time ≥12 days. Supplementary Figure 3 demonstrates the electropherograms obtained from the tissues fixed for a different time by using the two kits. The results have shown the superiority of the novel X-InDel kit to genotype severely degraded DNA in forensic casework.

### Balance of Peak Height

Ratios of the peak height of the alleles were all greater than 80% for each heterozygous locus. In addition, the peak height ratios of different loci within the same dye channel were 26, 45, 34, and 27% for blue, green, yellow, and red dye, respectively. Average RFU value for the blue dye was 5,379, 4,444 for green dye, 3,990 for yellow dye, and 3,303 for red dye.

### Size Precision

**FIGURE 3:**
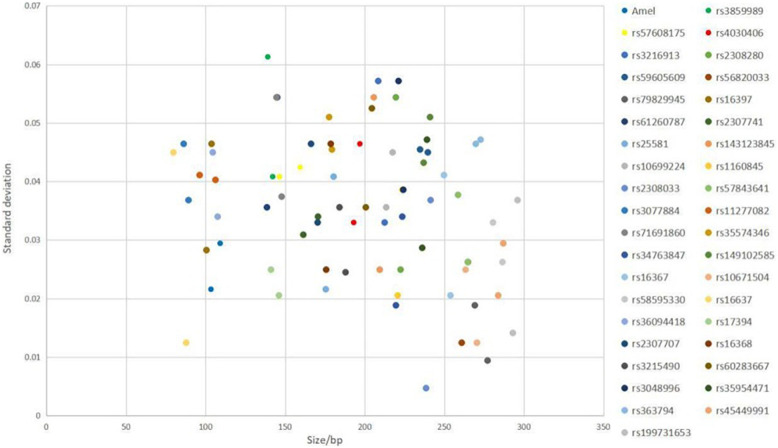
Size precision study of AGCU X-InDel 38 kit was performed on 3130XL Genetic Analyzer. The *X*-axis represents the fragment sizes of all allelic ladder samples at each locus, and the *Y*-axis represents the standard deviation of each allele size.

The results showed that the largest standard deviation observed was 0.061283 bases for rs3859989, and the lowest was 0.004714 bases for rs2308033 (Figure 3), which were well below the threshold size of 0.15 bases. Overall, the AGCU X-InDel 38 kit showed high allele size accuracy in allele detection.

### PCR Conditions

#### Reaction Components

In this study, successful amplification was demonstrated with the PCR conditions of 3–10 μmol/L of primers, 3/10–8/10 of reaction mix and all concentrations of C-Taq polymerase. Occasional allele dropouts were observed at the concentrations of InDel primers <3 μmol/L and the volume fraction of reaction mix <3/10. With increasing concentrations of those reaction components, allele peak height showed a trend of increasing obviously. However, excessive component concentration like primers ≥8 μmol/L or volume fraction of reaction mix ≥6/10, may lead to the occurrence of interference signals and poor balance performance of allele peak height. Thus, the recommended optimal conditions for the AGCU X-InDel 38 kit were 5 μmol/L, 5/10, and 0.3 μl in the same order above (Supplementary Figure 4).

#### Annealing Temperature

**FIGURE 4:**
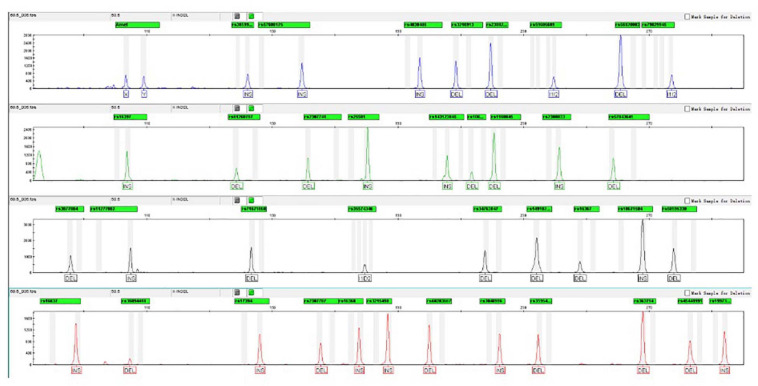
Electropherograms of control DNA 9948 amplified using the AGCU X-InDel 38 kit at 58.5°C.

After testing the annealing temperatures of 55.9, 56.5, 57.2, 57.9, 58.5, 59.2, 59.9, and 60.5°C, complete profiles were well observed for replicates. When the temperature dropped below 56.5°C, a marked non-specific peak was observed with an amplicon size of 258 bp. Moreover, the efficiency of amplification for partly tested loci was markedly affected by the increase in the temperature (≥59.2°C). In that case, the peak heights turned badly balanced, and rs56820033 showed an allelic dropout at 60.5°C. On consideration of perfect profiles and appropriate balance of peak height, 58.5°C was recommended to be the optimal annealing temperature. The electropherogram of control DNA 9948 amplified using AGCU X-InDel 38 kit at 58.5°C is displayed in Figure 4.

### Allele Frequency and Forensic Parameter Analysis

**FIGURE 5:**
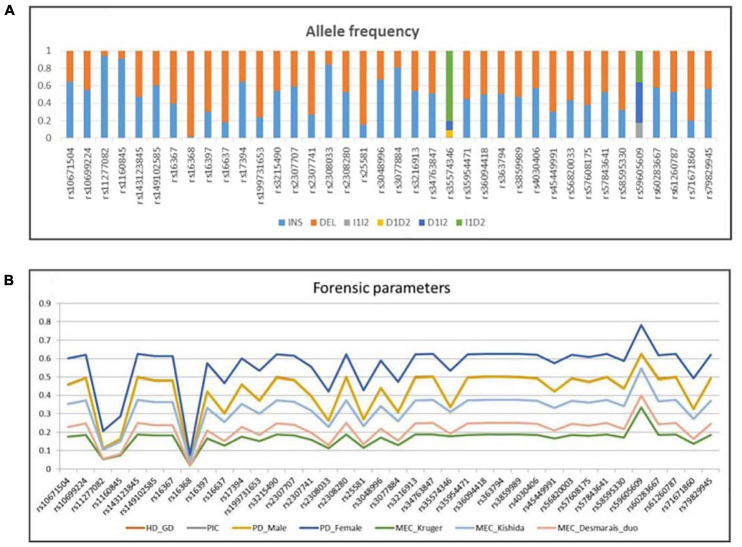
**(A)** Allele frequency and **(B)** forensic parameters of 38 X-InDel loci in a group of 200 unrelated males from Guangdong Han population.

The allele frequencies and forensic statistical parameters for AGCU X-InDel 38 kit are provided in Figure 5 and Supplementary Tables 2, 3. The PIC ranged from 0.03843168 at rs16368 locus to 0.546322699 at rs59605609 locus. The rs16368 locus had a minimum GD value of 0.039396985, and the rs59605609 locus had a maximum value of 0.626683417. Additionally, the pairwise LD analysis was conducted to reveal allelic association between 38 loci, and a total of 703 pairs were tested. After Bonferroni correction for multiple testing was applied to adjust threshold *p*-value (*p* ≤ 0.05/703), significant LD was observed at the following 12 pairs: rs11277082–rs16368, rs16637–rs2308033, rs1160845–rs25581, rs2307707–rs3048996, rs10671504–rs3077884, rs3215490–rs36094418, rs17394–rs4030406, rs2308280–rs57843641, rs45449991–rs58595330, rs143123845–rs59605609, rs363794–rs60283667, and rs35954471–rs71671860. The existence of LD may be the results of close physical location, since all markers are located on the same chromosome. In addition, population events like drift, selection, non-random mating, stratification, or admixture may also lead to LD (Hedrick, 1987; Medina-Acosta, 2011). The LD indicated the existence of non-random association of alleles of those pairwise loci existing at population-level. As recommended by the DNA commission of the ISFG, the pairwise loci must be analyzed together, and haplotype frequencies should be used for likelihood calculations when significant association is found among markers (Tillmar et al., 2017).

The values of discrimination power in males (PD_Male_) were in the range of 0.0392 (rs16368)–0.62355 (rs59605609), while those in females (PD_Female_) were in the range of 0.07609504 (rs16368)–0.78105809625 (rs59605609). The combined PD_Male_ and PD_Female_ were both more than 0.999999999. The MEC_Krüger_ varied between 0.01921584 and 0.3336460035 for the deficiency cases, MEC_Kishida_ between 0.03843168 and 0.54632269875 for the normal trios, and MEC_Desmarais  Duo_ between 0.0196000000000002 and 0.39966 for the duos. Meanwhile, the combined MECs were 0.999369481, 0.999999917, and 0.999941556, for the deficiency cases, normal trios, and duos, respectively. In this study, the combined PDs and MECs, calculated in the Guangdong Han group using AGCU X-InDel 38 kit, showed higher values than those in other X-InDel studies (Freitas et al., 2010; Pereira et al., 2012; Edelmann et al., 2016). The high forensic statistical parameters for AGCU X-InDel 38 kit demonstrated its powerful performance in human identification and kinship tests, even in some deficiency cases.

## Conclusion

In this study, a novel multiplex system for 38 X-InDels, mainly designed for the analysis of samples from East Asia, was developed for forensic human identification and kinship tests. The validation studies indicated that AGCU X-InDel 38 kit was sensitive, specific, robust, and highly informative. More importantly, due to high values of the PDs and MECs, the AGCU X-InDel 38 kit was significantly powerful in human identification and could be used as a complementary tool for kinship tests, especially in some deficiency cases.

## Data Availability Statement

The original contributions presented in the study are included in the article/Supplementary Material, further inquiries can be directed to the corresponding author/s.

## Ethics Statement

The studies involving human participants were reviewed and approved by the Biomedical Ethical Committee of the Southern Medical University. The patients/participants provided their written informed consent to participate in this study.

## Author Contributions

LC and YW performed the data analyses and wrote the manuscript. XP and WW performed the experiment. WD and XH helped perform the analysis with constructive discussions. CX contributed to the manuscript submission and revision. ZT conducted the collection of case samples and population samples. ChaoL and ChanL contributed to the conception of the study. All authors listed have made a substantial, direct and intellectual contribution to the work, and approved it for publication.

## Conflict of Interest

The authors declare that the research was conducted in the absence of any commercial or financial relationships that could be construed as a potential conflict of interest.

## Publisher’s Note

All claims expressed in this article are solely those of the authors and do not necessarily represent those of their affiliated organizations, or those of the publisher, the editors and the reviewers. Any product that may be evaluated in this article, or claim that may be made by its manufacturer, is not guaranteed or endorsed by the publisher.
