# Supplementary figures and images for: Development and Validation of a Forensic Multiplex System With 38 X-InDel Loci
Source: Front Genet. 2021 Aug 17;12:670482. doi: 10.3389/fgene.2021.670482 (PMC8416044; doi:10.3389/fgene.2021.670482)

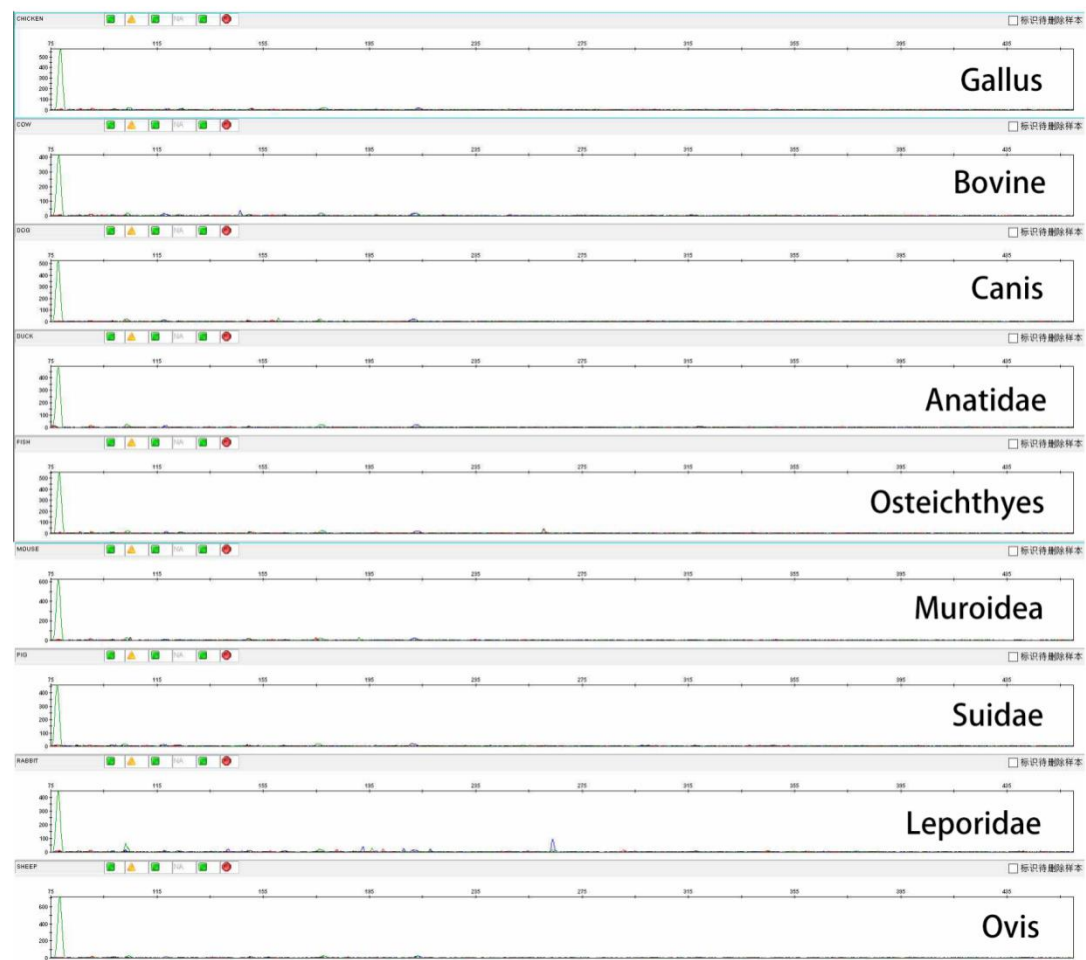

Figure S1. Electropherograms from species specificity studies of the AGCU X-InDel 38 kit.

Supplement: Supplementary file 1 [file Image_1.pdf]
