# Supplementary material for: Development and Validation of a Forensic Multiplex System With 38 X-InDel Loci
Source: Front Genet. 2021 Aug 17;12:670482. doi: 10.3389/fgene.2021.670482 (PMC8416044; doi:10.3389/fgene.2021.670482)
Supplement: Supplementary file 2 [file Image_2.pdf]

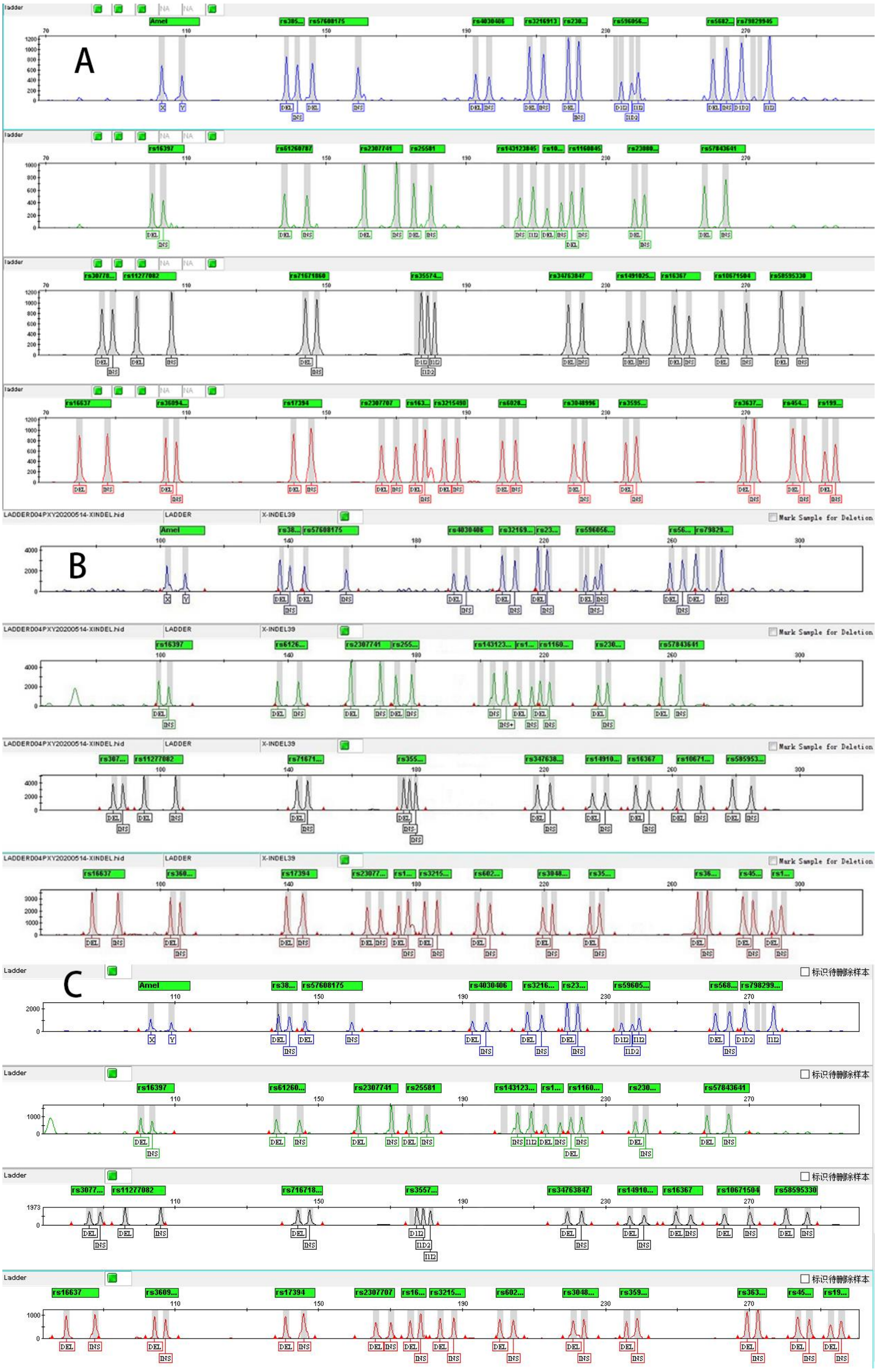

Figure S2. Electropherograms of allelic ladders genotyped with the AGCU X-InDel 38

kit in three separate laboratories, (A) Guangzhou Forensic Science Institute and (B, C) two of its branches.
