# Supplementary material for: Development and Validation of a Forensic Multiplex System With 38 X-InDel Loci
Source: Front Genet. 2021 Aug 17;12:670482. doi: 10.3389/fgene.2021.670482 (PMC8416044; doi:10.3389/fgene.2021.670482)
Supplement: Supplementary file 3 [file Image_3.pdf]

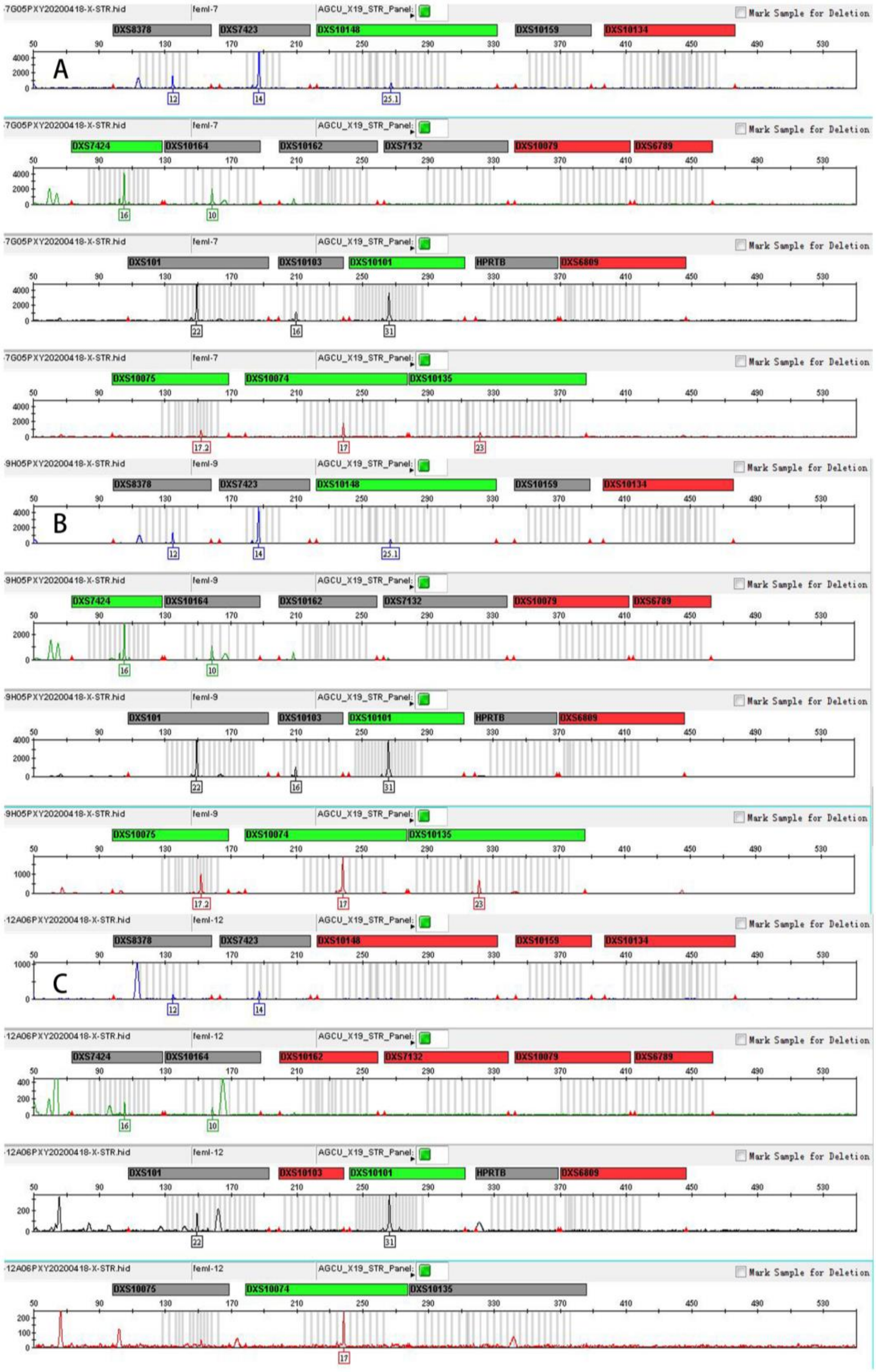
A
B
C
7G05PXY20200418-X-STR.hid
feml-7
9H05PXY20200418-X-STR.hid
feml-9
12A06PXY20200418-X-STR.hid
feml-12
AGCU_X19_STR_Panel
Mark Sample for Deletion
DXS8378
DXS7423
DXS10148
DXS10159
DXS10134
DXS7424
DXS10164
DXS10162
DXS7132
DXS10079
DXS6789
DXS101
DXS10103
DXS10101
HPRTB
DXS6809
DXS10075
DXS10074
DXS10135
12
14
25.1
16
10
22
31
17.2
17
23

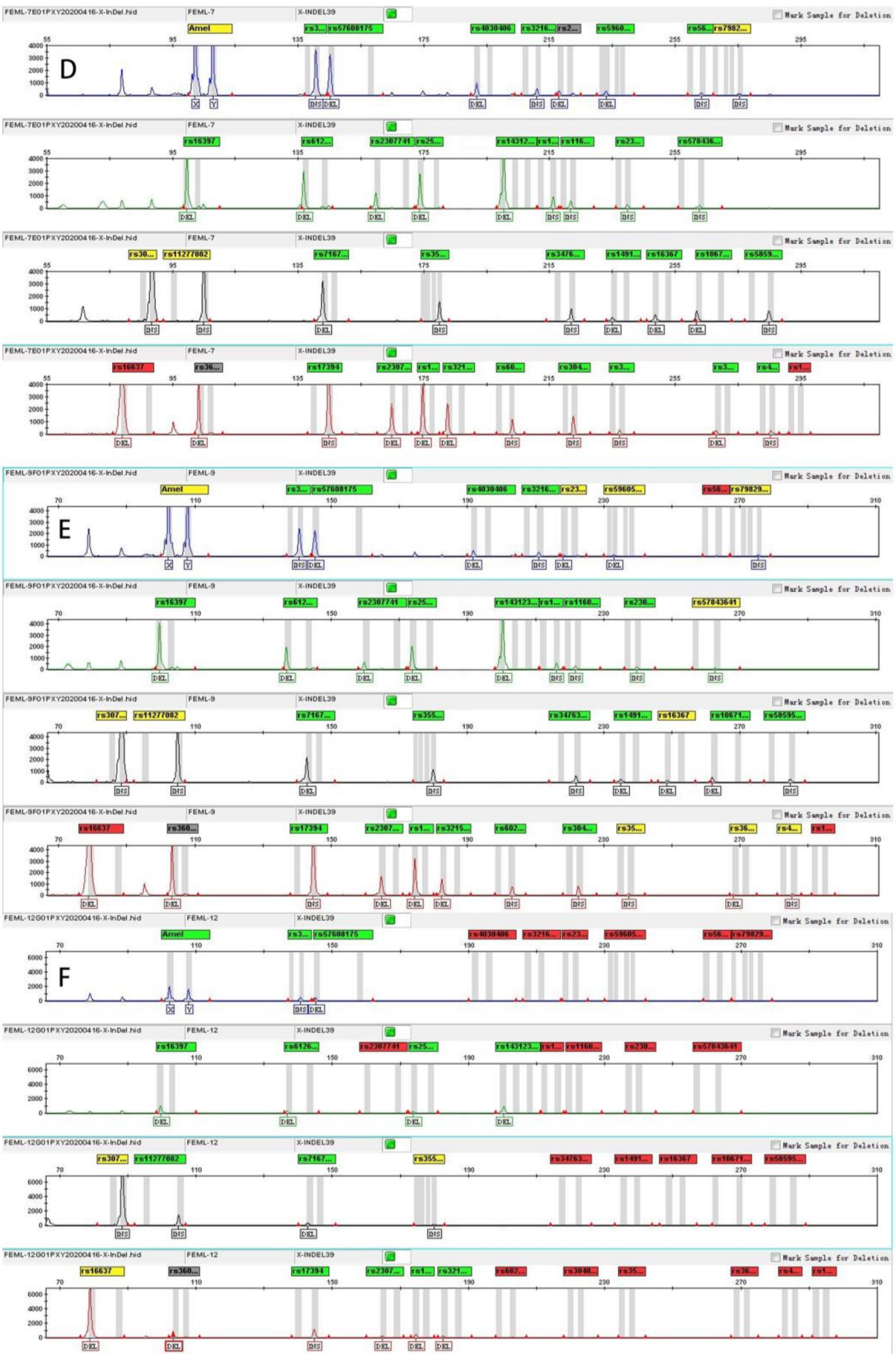

Figure S3. Electropherograms genotyped with (A, B, C) AGCU X19 STR kit and (D, E, F) AGCU X-InDel 38 kit from tissues for 7, 9 and 12 formalin soaked days, respectively.
