# Supplementary material for: Development and Validation of a Forensic Multiplex System With 38 X-InDel Loci
Source: Front Genet. 2021 Aug 17;12:670482. doi: 10.3389/fgene.2021.670482 (PMC8416044; doi:10.3389/fgene.2021.670482)
Supplement: Supplementary file 5 [file Table_1.docx]

Table S1. The numbers and ratios of detected loci in 56 case samples tested by the AGCU X19 STR kit and AGCU X-InDel 38 kit, respectively.

| **Sample No.** | **Sample Type** | **AGCU X19 STR kit** | | | **AGCU X-InDel 38 kit** | | |
| --- | --- | --- | --- | --- | --- | --- | --- |
|  |  | **detection number** | | **detection rate** | **detection number** | | **detection rate** |
| 1 | Blood stain1(13 years ago) | 10 | 52.63% | | 29 | 74.36% | |
| 2 | Blood stain2(13 years ago) | 14 | 73.68% | | 38 | 97.44% | |
| 3 | Blood stain3(13 years ago) | 9 | 47.37% | | 25 | 64.10% | |
| 4 | Blood stain4(13 years ago) | 10 | 52.63% | | 37 | 94.87% | |
| 5 | Blood stain5 | 17 | 89.47% | | 39 | 100.00% | |
| 6 | Blood stain6 | 18 | 94.74% | | 39 | 100.00% | |
| 7 | Blood stain7 | 19 | 100.00% | | 39 | 100.00% | |
| 8 | Straw1 | 19 | 100.00% | | 39 | 100.00% | |
| 9 | Straw2 | 19 | 100.00% | | 39 | 100.00% | |
| 10 | Straw3 | 18 | 94.74% | | 39 | 100.00% | |
| 11 | Toothbrush1 | 19 | 100.00% | | 39 | 100.00% | |
| 12 | Toothbrush2 | 19 | 100.00% | | 39 | 100.00% | |
| 13 | Toothbrush3 | 18 | 94.74% | | 39 | 100.00% | |
| 14 | Cigarette butt1 | 18 | 94.74% | | 39 | 100.00% | |
| 15 | Cigarette butt2 | 17 | 89.47% | | 39 | 100.00% | |
| 16 | Cigarette butt3 | 17 | 89.47% | | 39 | 100.00% | |
| 17 | Cigarette butt4(17 years ago) | 13 | 68.42% | | 35 | 89.74% | |
| 18 | Swab of bottleneck1 | 17 | 89.47% | | 39 | 100.00% | |
| 19 | Swab of bottleneck2 | 14 | 73.68% | | 36 | 92.31% | |
| 20 | Swab of bottleneck3 | 16 | 84.21% | | 38 | 97.44% | |
| 21 | Swab of bottleneck4 | 17 | 89.47% | | 39 | 100.00% | |
| 22 | Swab of bottleneck5 | 12 | 63.16% | | 35 | 89.74% | |
| 23 | Buccal swab(13 years ago) | 10 | 52.63% | | 22 | 56.41% | |
| 24 | Facial tissue1 | 9 | 47.37% | | 28 | 71.79% | |
| 25 | Facial tissue2 | 17 | 89.47% | | 39 | 100.00% | |
| 26 | Facial tissue3 | 16 | 84.21% | | 36 | 92.31% | |
| 27 | Facial tissue4(17 years ago) | 7 | 36.84% | | 22 | 56.41% | |
| 28 | Swab of charging cable1 | 14 | 73.68% | | 39 | 100.00% | |
| 29 | Swab of charging cable2 | 7 | 36.84% | | 18 | 46.15% | |
| 30 | Swab of charging cable3 | 18 | 94.74% | | 39 | 100.00% | |
| 31 | Swab of charging cable4 | 13 | 68.42% | | 36 | 92.31% | |
| 32 | Sealing tape1 | 12 | 63.16% | | 30 | 76.92% | |
| 33 | Sealing tape2 | 17 | 89.47% | | 39 | 100.00% | |
| 34 | Sealing tape3 | 10 | 52.63% | | 27 | 69.23% | |
| 35 | Swab of doorknob1 | 17 | 89.47% | | 39 | 100.00% | |
| 36 | Swab of doorknob2 | 7 | 36.84% | | 23 | 58.97% | |
| 37 | Swab of doorknob3 | 16 | 84.21% | | 38 | 97.44% | |
| 38 | Swab of stick1 | 12 | 63.16% | | 37 | 94.87% | |
| 39 | Swab of stick2 | 8 | 42.11% | | 28 | 71.79% | |
| 40 | Swab of stick3 | 8 | 42.11% | | 21 | 53.85% | |
| 41 | Swab of mobile phone1 | 18 | 94.74% | | 39 | 100.00% | |
| 42 | Swab of mobile phone2 | 18 | 94.74% | | 39 | 100.00% | |
| 43 | Swab of mobile phone3 | 17 | 89.47% | | 39 | 100.00% | |
| 44 | Swab of zipper head1 | 16 | 84.21% | | 36 | 92.31% | |
| 45 | Swab of zipper head2 | 7 | 36.84% | | 26 | 66.67% | |
| 46 | Swab of cable connector1 | 6 | 31.58% | | 13 | 33.33% | |
| 47 | Swab of cable connector2 | 8 | 42.11% | | 21 | 53.85% | |
| 48 | Swab of electronic scale | 17 | 89.47% | | 39 | 100.00% | |
| 49 | Swab of zip lock bag | 14 | 73.68% | | 36 | 92.31% | |
| 50 | Formalin soaked tissue1(0 day) | 19 | 100.00% | | 39 | 100.00% | |
| 51 | Formalin soaked tissue2(1 day) | 19 | 100.00% | | 39 | 100.00% | |
| 52 | Formalin soaked tissue3(3 days) | 19 | 100.00% | | 39 | 100.00% | |
| 53 | Formalin soaked tissue4(5 days) | 19 | 100.00% | | 39 | 100.00% | |
| 54 | Formalin soaked tissue5(7 day) | 11 | 57.89% | | 38 | 97.44% | |
| 55 | Formalin soaked tissue6(9 days) | 11 | 57.89% | | 37 | 94.87% | |
| 56 | Formalin soaked tissue7(12 days) | 7 | 36.84% | | 17 | 43.59% | |
