# Supplementary material for: Development and Validation of a Forensic Multiplex System With 38 X-InDel Loci
Source: Front Genet. 2021 Aug 17;12:670482. doi: 10.3389/fgene.2021.670482 (PMC8416044; doi:10.3389/fgene.2021.670482)
Supplement: Supplementary file 4 [file Image_4.pdf]

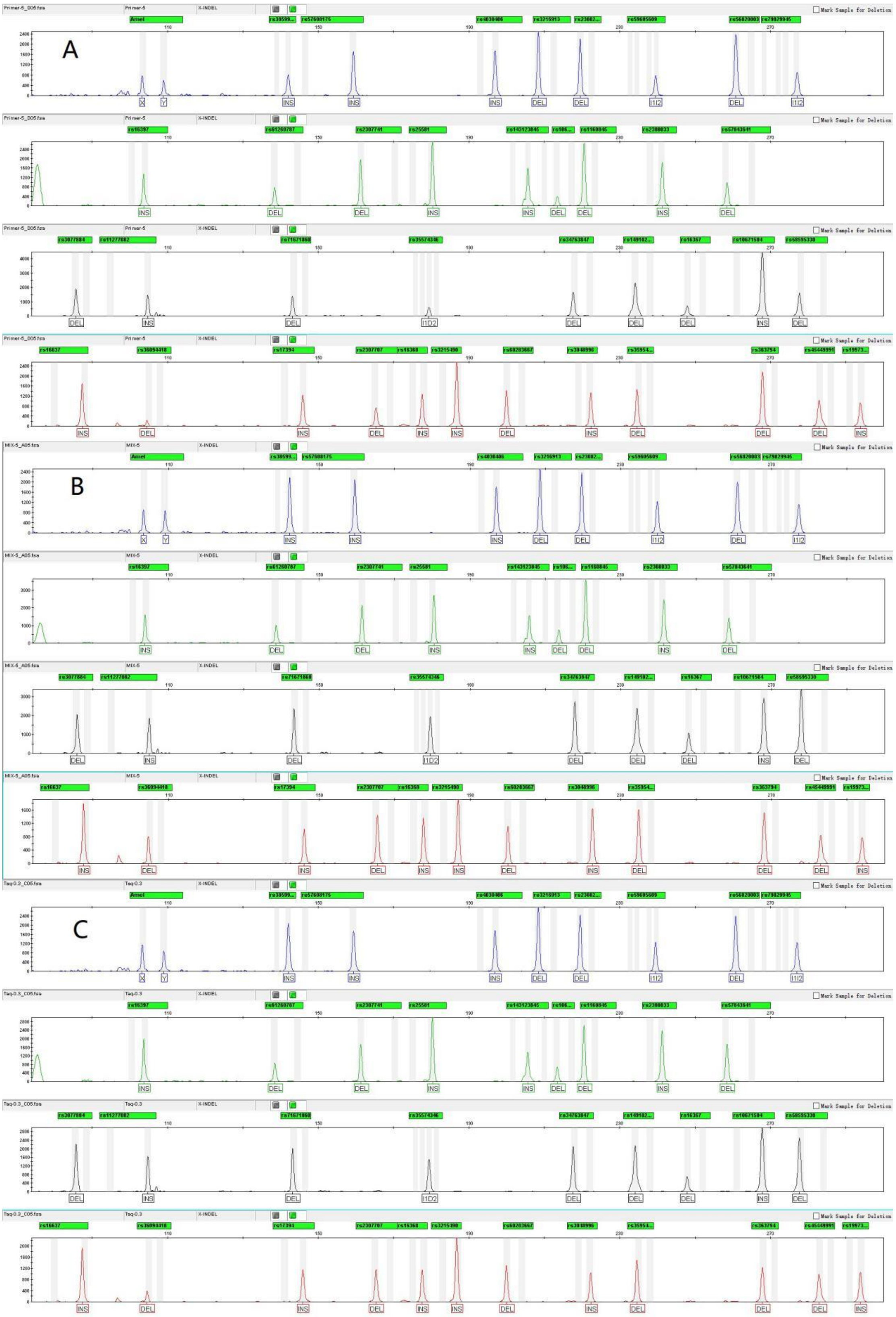

Figure S4. Electropherograms of amplified control DNA 9948 in the presence of (A) 5µM of the primer, (B) 5/10 of the reaction mix and (C) 0.3µL of C-Taq polymerase.
